# Genome Sequences of Two *Lactococcus garvieae* Strains Isolated from Meat

**DOI:** 10.1128/genomeA.00018-12

**Published:** 2013-01-15

**Authors:** Giovanni Ricci, Chiara Ferrario, Francesca Borgo, Giovanni Eraclio, Maria Grazia Fortina

**Affiliations:** Dipartimento di Scienze per gli Alimenti, la Nutrizione e l’Ambiente, Università degli Studi di Milano, Milan, Italy

## Abstract

*Lactococcus garvieae* is an important fish pathogen and an emerging opportunistic human pathogen, as well as a component of natural microbiota in dairy and meat products. We present the first report of genome sequences of *L. garvieae* I113 and Tac2 strains isolated from a meat source.

## GENOME ANNOUNCEMENT

*Lactococcus garvieae*, which was originally isolated from cows with mastitis (1), is one of the most important fish pathogens, responsible for lactococcosis, a bacterial disease that causes great economic losses in the aquaculture industry worldwide (2). In recent years, *L. garvieae* has gained recognition as an opportunistic human pathogen because it has been associated with an increasing number of human infections (3). Moreover, it has repeatedly been reported to be a major component of the native microbiota of dairy products manufactured from raw milk (4, 5). Thus, this bacterium has proved to be widely distributed, because it was also isolated from clinical specimens from other animal species (6, 7), from vegetables (8), and from meat (9, 10). Recently, the genome sequences of five *L. garvieae* fish-derived strains (11–14), along with those of two strains isolated from cheese (14, 15) and one strain isolated from human blood (16), have been released. However, although this bacterium appears to be very widespread in the meat environment (17), no sequenced *L. garvieae* genomes from a meat source exist.

We report the draft genome sequences of *L. garvieae* I113, isolated from Italian pork sausage, and *L. garvieae* Tac2, isolated in Italy from turkey meat. The whole-genome shotgun sequencing was performed with Illumina sequencing technology. Quality-filtered reads were assembled into contigs using CLC Genomics Workbench version 5.1.0. Functional annotation was performed by the RAST (Rapid Annotation using Subsystem Technology) server (18) and checked by BLAST analysis (19) when needed.

The draft genome sequence of *L. garvieae* I113 includes 49 contigs covering 2,178,733 bases with a G+C content of 37.9%. A total number of 2,124 predicted coding sequences (CDSs) were annotated. The draft genome sequence of *L. garvieae* Tac2 includes 97 contigs covering 2,242,863 bases with a G+C content of 38.2%, and it contains 2,153 CDSs. The draft genome sequences of the I113 and Tac2 strains contain 310 and 302 subsystems (sets of related functional roles) according to the RAST server, respectively. We used this information to reconstruct the metabolic network.

A preliminary comparative genome analysis with the *L. garvieae* sequences previously published revealed the presence of new genes, including *tet*(S) and several phage-related genes present exclusively in the I113 strain. Recently, in the human clinical strain *L. garvieae* 21881, five plasmids named pGL1 to pGL5, harboring genes encoding bacteriocins and putative virulence factors, were characterized (20). Interestingly, the sequence of pGL2 was present in the I113 strain, and the sequences of pGL1 and pGL5 were present in the Tac2 strain with >95% sequence identity. The observed similarity suggests the existence of horizontal gene transfer events among *L. garvieae* strains.

The addition of other sequenced strains isolated from various environments can expand our knowledge about intraspecific genetic variation of this ubiquitous bacterium, especially that focused on the identification of genes potentially involved in virulence.

### Nucleotide sequence accession numbers. 

These Whole Genome Shotgun projects have been deposited in DDBJ/EMBL/GenBank under accession numbers AMFD00000000 (*L. garvieae* I113) and AMFE00000000 (*L. garvieae* Tac2). The versions described in this article are the first versions, with DDBJ/EMBL/GenBank accession numbers AMFD01000000 and AMFE01000000.
